# 非小细胞肺癌中MTAP蛋白及基因表达水平的检测

**DOI:** 10.3779/j.issn.1009-3419.2011.02.09

**Published:** 2011-02-20

**Authors:** 莎莎 李, 义军 张, 洪利 李, 保清 丁

**Affiliations:** 1 261053 潍坊，潍坊医学院药理学教研室 Department of Pharmacology, Weifang Medical College, Weifang 261053, China; 2 261053 潍坊，潍坊医学院医学研究实验中心 Medicine Research Experiment Center of Science Research, Weifang Medical College, Weifang 261053, China

**Keywords:** 甲硫腺苷磷酸化酶, 肺肿瘤, 免疫组织化学, 实时定量PCR, MTAP, Lung neoplasms, Immunohistochemistry, Real-time quantitative PCR

## Abstract

**背景与目的:**

*MTAP*基因是一种肿瘤抑制基因，在多种肿瘤组织中存在表达异常现象。本研究旨在探讨MTAP在非小细胞肺癌（non-small cell lung cancer, NSCLC）组织、癌旁组织以及边缘肺组织中的表达水平及临床意义。

**方法:**

应用免疫组织化学方法及IPP图像分析软件观察52例NSCLC患者组织中MTAP蛋白的表达，采用实时荧光定量PCR及2^-ΔΔCt^法定量分析MTAP mRNA的相对表达量。

**结果:**

MTAP在NSCLC组织中的蛋白表达分别明显低于癌旁组织和边缘肺组织（t分别为10.283、10.940，均*P*＜0.001），MTAP蛋白的表达与肺癌患者性别、年龄、吸烟史以及肿瘤的病理类型无明显关系，但与肿瘤的分化程度有密切关系（*t*=2.310, *P*=0.025）。NSCLC组织中MTAP mRNA相对表达量明显低于癌旁组织（*t*=9.902, *P*＜0.001）。

**结论:**

肺癌组织中存在MTAP mRNA和蛋白表达水平下调，可能与NSCLC的发生和进展有关。

## Abstract (en)

**Background and objective:**

The abnormal expression of MTAP, a tumor suppressor gene, is found in a variety of tumor tissues. The aim of this study is to detect the expression of MTAP mRNA protein and the clinical significance for the therapy of non-small cell lung cancer tissue (NSCLC).

**Methods:**

The expression of MTAP protein was detected by immunohistochemistry in 52 cases of NSCLC patients. The relative expression MTAP mRNA was detected by real-time quantitative PCR.

**Results:**

The expression of MTAP protein in NSCLC tissue was significantly lower than that in paracarcinomous tissue and borderline lung tissue respectively (*t*=10.283, 10.940, *P* < 0.001). There was no significant difference among gender, age, smoking history, histology except differentiation (*t*=2.310, *P*=0.025). The MTAP mRNA relative expression in NSCLC tissue was significantly lower than that in paracarcinomous tissue (*t*=9.902, *P* < 0.001).

**Conclusion:**

Downregulation of MTAP protein and gene expression is correlated to the oncogenesis and progression of NSCLC.

非小细胞肺癌（non-small cell lung cancer, NSCLC）在肺癌中常见，占其总数的80%-85%，主要包括鳞癌（squamous cell carcinoma, SCC）、腺癌（adeno carcinoma, AC）和大细胞癌（large cell cancer, LCC）3种亚型。随着近10年来在肿瘤诊断、手术、放化疗等方面的研究取得的一些进步，乳腺癌、前列腺癌等患者的5年生存率得到明显提高，但NSCLC患者的5年生存率仍较低，在发展中国家不足9%。

甲硫腺苷磷酸化酶（methylthioadenosine phosphorylase, MTAP）是嘌呤和甲硫氨酸合成补救途径中的一个关键酶，催化MTA生成ATP、dAMP和甲硫氨酸，参与细胞的能量合成与蛋白合成。MTAP在正常细胞和组织中表达丰富，但在多种恶性细胞系和肿瘤组织中都存在高频缺失或降低。有文献^[1]^报道，MTAP在NSCLC中存在纯合性缺失。本研究通过检测NSCLC组织、癌旁组织、边缘肺组织中MTAP的表达水平，结合临床资料探讨NSCLC中MTAP的表达变化及与其临床病理特征的关系，并为NSCLC的临床诊断、治疗提供新的线索。

## 1 材料与方法

### 1.1 材料

#### 1.1.1 实验标本

原发NSCLC患者52例，2008年1月-2009年12月于潍坊市人民医院和潍坊医学院附属医院行外科手术治疗，收集手术时切下的新鲜标本。根据所取组织分为NSCLC组、癌旁组（距癌边缘2 cm）、边缘组（距癌边缘5 cm以上的边缘肺组织），手术切除的新鲜组织以冻存管分装后立即置入液氮罐中，后转入-70 ℃冰箱保存。其中男性39例，女性13例；年龄43岁-72岁，中位年龄60.8岁；有吸烟史者38例，无吸烟史者14例；鳞癌28例，腺癌24例；高-中分化40例，低分化12例。所有病例术前均未采取放疗、化疗和免疫治疗。患者均知情同意。

#### 1.1.2 主要试剂

Trizol^®^ Reagent、QuantiTect SYBR Green PCR Kit购自Invitrogen公司；AMV第一链cDNA合成试剂盒购自上海生工生物有限公司；兔抗人MTAP多克隆抗体购自武汉三鹰生物有限公司；SP免疫组化检测试剂盒购自北京中杉金桥生物技术有限公司。

### 1.2 方法

#### 1.2.1 免疫组织化学法检测MTAP的表达

所有组织经10%甲醛固定，常规石蜡包埋，每个蜡块行5 μm厚连续切片，HE染色。观察组织病理结构，由病理科医师做出诊断，且癌旁组未见癌细胞。应用免疫组化SP法染色：以PBS代替一抗为阴性空白对照，已知结肠癌阳性切片为阳性对照；切片组织常规脱蜡入水后，微波抗原修复10 min，一抗孵育4 ℃过夜，DAB显色5 min，苏木素复染。

#### 1.2.2 图像分析

在显微镜相同光强度下，数码相机关掉自动白平衡等功能，拍摄图片（×200）。每例组织切片随机采取5个视野，应用Image-Pro Plus 6.0（IPP 6.0）专业图像分析软件对所采集的图片进行图像分析，选取AOI计算平均光密度（mean optical density, MOD）值，并在同一参数条件下对所有图片进行MOD值分析。

#### 1.2.3 实时荧光定量PCR检测MTAP mRNA

Trizol试剂盒提取NSCLC组织、癌旁组织以及边缘肺组织中的总RNA，逆转录合成cDNA。根据Gene Bank中MTAP的mRNA序列，利用Primer Premier 5.0引物设计软件，设计引物序列，实时荧光定量PCR检测样本中MTAP cDNA水平，*β*-*actin*基因做内对照。引物：MTAP（292 bp）：上游引物5'-CGCTTGGTTCCCTTAGTC-3'，下游引物5'-GATGTTCGCCTGGTAGTT-3'；β-actin（314 bp）：上游引物5'-TCCTGTGGCATCCACGAAACT-3'；下游引物5'-GAAGCATTTGCGGTGGACGAT-3'。反应在25 μL的体系中进行，其中2×QuantiTect SYBR Green PCR 12.5 μL、10 μM的上下游引物各1 μL、样本cDNA 2 μL（300 ng）、RNA酶灭活水8.5 μL。热循环如下：94 ℃、5 min；94 ℃、30 s，57 ℃、30 s，72 ℃、60 s，40个循环；72 ℃、10 min。应用2^-ΔΔCt^法^[2]^分析目的基因和内参基因的Ct值，计算NSCLC组织和癌旁组织中MTAP mRNA相对于边缘肺组织的表达差异。

### 1.3 统计学分析

采用SPSS 13.0软件分析数据，结果用Mean±SD表示，组间比较采用独立样本*t*检验，*P*＜0.05为差异有统计学意义。

## 2 结果

### 2.1 NSCLC组织、癌旁组织、边缘肺组织中MTAP的免疫组织化学检测

各组肺组织切片MTAP蛋白免疫组织化学染色（图 1）胞浆着色，呈棕黄色或黄褐色颗粒状。52例NSCLC组织中有3例MTAP阴性，49例MTAP阳性，阴性率为（5.8%），而在癌旁组织、边缘肺组织中均有表达。图像MOD值分析显示，NSCLC组MTAP蛋白含量为0.275±0.142，癌旁组为0.495±0.059，边缘组为0.501± 0.028。统计学分析显示NSCLC组的MTAP蛋白表达水平明显低于癌旁组和边缘组（*t*分别为10.283、10.940，均*P*＜0.001），而癌旁组与边缘组之间无明显差异（*t*=0.694, *P*=0.491）。MTAP蛋白的表达与性别、年龄、吸烟史以及肿瘤的病理类型无明显相关，但与肿瘤的分化程度有关（*t*=2.310, *P*=0.025）（表 1）。

**1:**
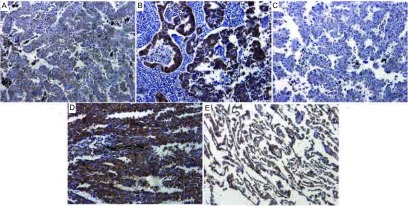
各组肺组织MTAP蛋白表达的免疫组化染色结果（×200）。A、B：NSCLC组织中（鳞癌、腺癌）MTAP的阳性表达；C：NSCLC组织中MTAP的阴性表达；D：癌旁组织中MTAP的阳性表达；E：边缘肺组织中MTAP的阳性表达。 Immunohistochemical staining results for MTAP in lung tissue (×200). A: Positive expressions of MTAP in squamous cell carcinoma; B: Positive expressions of MTAP in ademo carcinoma; C: Negative expression of MTAP in NSCLC tissue; D: Positive expression of MTAP in paracarcinomous tissue; E: Positive expression of MTAP borderline lung normal tissue.

**1:** MTAP在NSCLC组织中的表达与患者临床病理特征的关系（Mean±SD） The relationship between expression of MTAP and clinicopathological variables in NSCLC tissue (Mean±SD)

Group	*n*	MOD	*t*	*P*
Gender			0.298	0.767
Male	39	0.285±0.117		
Female	13	0.279±0.043		
Age (years)			1.834	0.078
≤60	23	0.236±0.182		
> 60	29	0.313±0.081		
Smoking history			0.224	0.824
Ever	38	0.278±0.026		
Never	14	0.268±0.053		
Histology			0.119	0.906
Squamous cell carcinoma	28	0.273±0.118		
Adeno carcinoma	24	0.278±0.162		
Differentiation			2.310	0.025
Well-moderately differentiated	40	0.250±0.138		
Poorly differentiated	12	0.351±0.093		

### 2.2 NSCLC组织、癌旁组织、边缘组织中MTAP mRNA的表达

52例NSCLC组织、癌旁组织、边缘肺组织中均有表达（图 2），以边缘组作为对照组，MTAP mRNA相对表达量用以下公式计算：folds＝2^-ΔΔCt^，其中ΔΔCt ＝（Ct_MTAP_-Ct_β-actin_）_样品组_-（Ct_MTAP_-Ct_β-actin_）_对照组_，通过计算得出NSCLC组织相对于边缘肺组织的表达量为0.029 ±0.016；癌旁组织相对于边缘肺组织的表达量为0.117± 0.060，统计学分析显示NSCLC组织中MTAP mRNA相对表达量明显低于癌旁组织（*t*=9.902, *P*＜0.001）。

**2:**
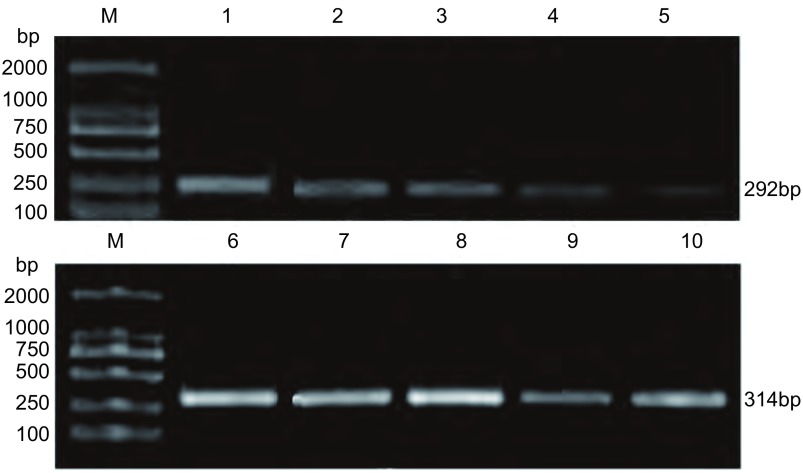
各组组织中*MTAP*基因PCR产物电泳图。M：DNA marker（DL 2000）；第1-5泳道：MTAP mRNA的表达；第6-10泳道：*β*-actin mRNA的表达；第1、2、6、7泳道：边缘正常组织；第3、4、8、9泳道：癌旁组织；第5、10泳道：NSCLC组织。 PCR analysis of MTAP mRNA in NSCLC tissue. Lane M: DNA marker (DL 2000); Lane 1-5: Expression of MTAP mRNA; Lane 6-10: Expressions of *β*-actin mRNA; Lane 1, 2, 6, 7: borderline normal tissue; Lane 3, 4, 8, 9: paracarcinomous tissue; Lane 5, 10: NSCLC tissue.

## 3 讨论

*MTAP*基因定位于9p21，与CDKN2A、CDKN2B以及ARF临近，该区域基因的缺失或超甲基化可导致基因表达的缺失。在恶性黑色素瘤^[3]^、蕈样肉芽肿病^[4]^、腹膜间皮瘤^[5]^等发现，9p21的大片区域纯合性缺失导致CDKN2A、ARF、MTAP失活，该部位的缺失与肿瘤的预后密切相关^[4]^，并且CDKN2A、ARF参与细胞周期的调控^[6]^。但对于淋巴瘤的研究^[7]^发现*MTAP*是一种独立于CDKN2A、ARF的抑癌基因。

MTAP在多胺代谢以及腺嘌呤、甲硫氨酸的合成补救过程中发挥重要作用。MTAP催化MTA转化为腺嘌呤、MTR1P，并最终生成AMP、甲硫氨酸。在MTAP阴性的肿瘤细胞中，嘌呤合成主要依靠从头合成途径。在体外研究中，Krasinskas等^[8]^发现5’-脱氧-5’-甲硫腺苷与腺嘌呤类似物联合疗法可以杀死MTAP阴性的A549肺癌细胞，而对MTAP阳性的人成纤维细胞无作用。这提示MTAP可以用于肺癌的选择性化疗。MTAP的底物MTA是氨丙基转移酶、甲基转移酶的有效抑制剂^[9]^。Steven等^[10]^研究发现，*MTAP*基因缺失的黑色素瘤细胞内出现MTA四倍浓度的积聚，MTA介导成纤维细胞分泌基本的成纤维细胞生长因子（basis fibroblast growth factor, bFGF）和基质金属蛋白酶3（matrix metalloproteinase 3, MMP3），并与黑素瘤细胞、成纤维细胞中转录激活蛋白-1（activator protein-1, AP-1）活性上调有关，MTA诱导肿瘤细胞侵袭力增强。通过比较基因组杂交^[11]^发现，156例胃肠道间质瘤中有25例出现MTAP纯合性缺失，并且这种缺失与肿瘤的大小、有丝分裂速度、Ki-67指数、危险水平均呈正相关。MTAP的这些生物学特点提示其可能参与细胞的增殖和分裂以及肿瘤的侵润、转移等，因此被认为是肿瘤诊断和治疗的新的分子靶点。本研究取材于NSCLC组织、癌旁组织以及边缘肺组织，以研究*MTAP*基因在NSCLC不同发展阶段的表达情况及其相关性。免疫组化结果显示，MTAP在NSCLC组织、癌旁组织以及边缘肺组织中均有表达，但在NSCLC组织中的表达量明显低于癌旁组织以及边缘肺组织，说明MTAP通过改变肿瘤细胞的生长代谢参与了NSCLC的发生发展过程。

以往研究^[12]^表示，卵巢癌组织Western印迹检测到的MTAP蛋白缺失率高于RT-PCR检测到的MTAP mRNA的缺失率。本研究发现，通过免疫组织化学检测有3例NSCLC组织出现MTAP蛋白缺失，而PCR检测未发现MTAP mRNA的缺失，这种蛋白水平与基因水平检测结果的不同有异于以往研究结果^[13]^。其原因可能是，DNA甲基化程度改变导致的基因渐生性沉默、组蛋白尾部的共价修饰以及染色质变性，microDNA这些因素导致了实时定量PCR检测实体瘤中MTAP缺失的局限性^[14]^，这些发现表示，在诊断NSCLC患者的MTAP表达缺失时，免疫组织化学优于PCR。本研究通过免疫组织化学染色方法检测到，在高-中分化的NSCLC组织中MTAP蛋白表达显著下调，说明MTAP不但与NSCLC的发生发展有关，而且与肿瘤的恶性程度有关。在mRNA水平，NSCLC组织中*MTAP*基因相对表达量明显低于癌旁组织，表明在NSCLC发展过程中存在*MTAP*基因在转录水平的下调。MTAP蛋白的表达与患者性别、年龄、吸烟史以及肿瘤的病理类型无明显相关，这也可能与样本量有限相关，还需进一步加大样本量验证MTAP在转录和翻译水平的表达差异。
